# Mate choice for body size leads to size assortative mating in the Ryukyu Scops Owl *Otus elegans*

**DOI:** 10.1002/ece3.9578

**Published:** 2022-12-12

**Authors:** Akira Sawada, Tetsuya Iwasaki, Kana Akatani, Masaoki Takagi

**Affiliations:** ^1^ Biodiversity Division National Institute for Environmental Studies Tsukuba Japan; ^2^ Department of Biology and Geosciences, Graduate School of Science Osaka City University Osaka Japan; ^3^ Department of Natural History Science, Graduate School of Science Hokkaido University Sapporo Japan

**Keywords:** assortative mating, body size, island, *Otus elegans*, Ryukyu Scops Owl, sexual size dimorphism

## Abstract

Understanding evolutionary phenomena that involve size assortative mating requires elucidating the generating mechanisms on which assortment is based. Although various mechanisms have been suggested, their relative importance may differ across taxonomic groups. Males selecting for large, fecund females combined with the dominance of large males in the competition for females has been suggested as a major mechanism in specific groups. However, raptors do not appear to conform to this, because the selection for smallness among males (assumed in a theory of reversed sexual size dimorphism) and the selection for largeness among males (assumed in the theory of size assortative mating) are in opposite directions. We studied the assortative mating during a long‐term study of the Ryukyu Scops Owls *Otus elegans interpositus*. Significant assortative mating was found for culmen length (from the base to the tip of the bill) and wing length (from the bend of the wing to the tip of the longest primary). Statistical control of the spatial and temporal accessibility of potential mates did not affect the assortment. Males with short wings had slightly higher fitness components than those with long wings, and females settling early tended to have small wings. Considering that early‐settling females can preferentially choose their mates, these results suggest that smaller females have an advantage when choosing smaller males with good reproductive performance. Improved flying and hunting ability of smaller individuals may be the background of choosing smaller individuals. We propose that, not passive process like similarity between individuals and their potential mates, but active mate choice for small individuals is an explanation for the assortative mating in this owl.

## Graphical abstract

We investigated the cause of size assortative mating in a population of owl which shows reversed sexual size dimorphism. Results suggested that the cause was “small female's advantage in acquiring small males with good reproductive performance.” It enables generalization of traditional explanation for size assortative mating: “large male's advantage in acquiring large females with good fecundity,” which was based on the studies on organisms with non‐reversed sexual size dimorphism.

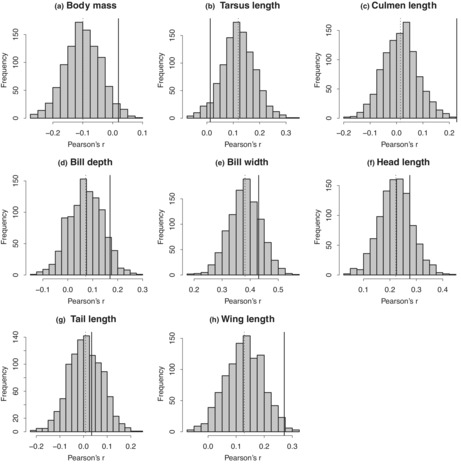

## 1 INTRODUCTION

Mated‐pair members often share phenotypic traits, indicative of assortative mating (Jiang et al., 2013). These traits include characteristics such as: coloration, body size, aggressiveness, genotype, metabolic state, and intelligence (reviewed in Jiang et al., 2013; Luo, 2017; Wang et al., 2019). Because assortative mating can be an incipient process of speciation, or assumed to be a prerequisite of speciation models (Bolnick & Fitzpatrick, 2007; Coyne & Orr, 2004; Elmer et al., 2009), and because it can be an outcome of sexual selection (Crespi, 1989; Wang et al., 2019), consequences of assortative mating have great significance for evolutionary biology. To accurately understand evolutionary phenomena involving assortative mating, the generating mechanisms of the assortments are also of importance (Galipaud et al., 2013). Here, we will use the term “assortative mating” to mean the circumstance where the phenotypic correlation between members of mated‐pair can be observed, irrespective of the mechanisms on which the correlation occurs, for simplicity. Focusing on size assortative mating among avian species, Wang et al. (2019) classified the mechanisms into three categories: (1) like meets like, (2) become alike, and (3) mate choice. We will review the three mechanisms in the following three paragraphs.

Mechanism 1: “like meets like” explains the correlation between paired individuals by resemblance between individuals and their potential mates (Wang et al., 2019). For example, potential mates nearby may have similar genotypes and phenotypes (Erlandsson & Rolán‐Alvarez, 1998; Indykiewicz et al., 2017). Under such circumstance, members of a pair resemble each other even without preference to similar individuals. Such similarity also arises from temporal separation of individuals (Hendry & Day, 2005). For lifelong monogamous species, potential mates for young recruits into the breeding population are often also young recruits. As young recruits of raptors often breed later than adults in a breeding season (Warkentin et al., 1992), this leads to assortment for age. If body size differs with age, then correlation of body size may occur as a consequence of age‐related temporal assortment (Wagner, 1999).

Mechanism 2: “become alike” explains the correlation between paired individuals by sharing the same environmental effect between mates (Wang et al., 2019). For species in which mates share resources such as territory and food, mates may resemble each other because they feed on similar food, use similar habitat, and are affected by similar environmental effects (Class & Brommer, 2018). Such similarity is expected to find in labile traits such as wing length and body mass.

Mechanism 3: “mate choice” explains the correlation between paired individuals by choice (or preference) by one or both mates (Wang et al., 2019). If the choice is based on the similarity, then positive correlation between the mates occurs. However, even without such a preference for similarity, correlation can arise. For example, if males prefer large females for reasons of their fecundity, and if large males are at an advantage in the competition for large females, then such competition results in pairs of large (competitive) males and large (fecund) females, and their opposites small (uncompetitive) males with small (infecund) females (Crespi, 1989). Hereafter, we call this the competition‐based mechanism. Note that the example above assumes only males' preference to females. Therefore, mutual choice is not unnecessary to the correlation among pair members to occur.

Although three mechanisms have been suggested irrespective of taxonomic groups, their relative importance may differ across groups. As previously mentioned, a competition‐based mechanism assumes that acquiring large fecund females is an advantage for males. In a review of the mechanisms involved in size assortative mating, Crespi (1989) suggested that such a competition‐based mechanism is dominant among arthropods. However, it may be less important for taxonomic groups with small variations in fecundity, or among groups with little correlation between female body size and fecundity (Pincheira‐Donoso & Hunt, 2017), since sufficient variation within the target of choice is necessary for such choice to work (Lehmann et al., 2007). In addition, the competition‐based mechanism may also be less important among species in which female choice is more important than male–male physical competition for females, as is suggested by research into anuran amphibians (Green, 2019). Considering these, if size assortative mating were to occur in an avian species, what might be the contributory mechanisms? Birds lay far fewer eggs than do arthropods, hence birds seem to have low inter‐individual variation in fecundity, and they are traditional subjects for studies of female choice of males. Assortative mating in birds may arise due to them having a different set of contributing mechanisms from other taxonomic groups (Wang et al., 2019).

Hawks, eagles, falcons, and owls (hereafter called “raptors,” for simplicity) offer interesting opportunities for investigating the cause of size assortative mating. Firstly, raptors are long‐lived and often show life‐long monogamy (König & Weick, 2008; McDonald et al., 2005). Such characteristics call for careful mate choice because it can greatly influence life‐time reproductive success (Wojczulanis‐Jakubas et al., 2018). Secondly, raptors occur worldwide and vary considerably in body size (Schoenjahn et al., 2020). This facilitates comparative analysis of the relationships between size assortative mating and various factors. Thirdly, female raptors are typically larger than males (reversed sexual size dimorphism: Mueller, 1986; Owens & Hartley, 1998; Krüger, 2005). One major hypothesis explaining the evolution of this dimorphism is the small‐male hypothesis, which considers that males are selected to be small thereby improving their agility, maneuverability, and foraging efficiency (Hakkarainen et al., 1996; Krüger, 2005). Intriguingly, this selection for smallness is the exact opposite of the selection for largeness which is assumed in competition‐based mechanism for size assortative mating. Furthermore, one of alternative hypotheses explaining the dimorphism relies on the intersexual size difference having been selected to reduce intersexual competition (Krüger, 2005; Pande & Dahanukar, 2012, see also Mueller, 1986 for other alternative explanations of the dimorphism). If this is the case, then dissimilarity in the size of mates (disassortative mating) rather than similarity (assortative mating) seems to be preferred. Based on these considerations, the occurrence of size assortative mating per se among raptors is interesting since it indicates coexistence of two selection pressures in different directions. Therefore, the underlying mechanism of the assortment is worth investigating.

Here, we address the existence of assortative mating and the generating mechanism of it in the Ryukyu Scops Owl *Otus elegans interpositus*, a species in which males are slightly smaller than females (Sawada, Iwasaki, Matsuo, et al., 2021). During the long‐term (since 2002) monitoring of an isolated population of this taxon, data have been accumulated on the morphology, reproductive success, territories, and age of breeding pairs. The aims of this study are (1) to describe size assortative mating, (2) investigate the possible mechanisms contributing to the detected mating patterns, referring to previously documented three mechanisms: “like meets like,” “become alike,” and “mate choice.”

## 2 MATERIALS AND METHODS

### 2.1 Material

*Otus elegans interpositus* is endemic to Minami‐daito, a small, isolated, oceanic island in Japan (Ornithological Society of Japan, 2012). The population on the island consists of 200–300 pairs, and their breeding activity and survival history have been studied annually since 2002 (Sawada et al., 2019; Takagi et al., 2007; Takagi, 2020). The owls are monogamous and pair‐bonds last, in most cases, until one of the pair dies (Akatani, 2011). Extra‐pair copulation occurs, but is uncommon (Sawada et al., 2020). Pairs maintain their territories throughout the year and tend to use the same nest sites in successive years (Akatani et al., 2011). Females lay a clutch of one to four eggs from mid‐March to mid‐May (Akatani et al., 2011; Sawada & Iwasaki, unpublished data; Takagi et al., 2007). The incubation and nestling periods each last about 1 month (Akatani et al., 2011; Sawada & Iwasaki, unpublished data; Takagi et al., 2007). Males carry food to their mates until the middle of the nestling period, and thereafter the parents share feeding duties (Murakami et al., 2022; Takagi & Akatani, 2011). There is no significant sexual difference in annual survival rate (Sawada, Iwasaki, Inoue, et al., 2021). The average body mass of adult males and adult females are 88.4 and 92.2 g, respectively, showing slight reversed sexual size dimorphism (Sawada, Iwasaki, Matsuo, et al., 2021).

### 2.2 Breeding monitoring

Since 2002 nests in natural cavities and nest boxes have been visited regularly to obtain data on breeding success. In this study, we have used data from 285 breeding attempts by 159 unique pairs consists of 138 individuals (some individuals bred multiple times), which were neither predated nor abandoned and for which we have complete data on the identity of the parents, egg laying data, and number of fledglings (Table S1). All chicks were ringed and measured, and blood samples were collected from them. Detailed field procedures have been described elsewhere (Akatani et al., 2011; Sawada et al., 2020; Takagi et al., 2007).

### 2.3 Territory identification

All territorial owls on the island have been recorded as part of mark‐recapture (mark‐resight) surveys since 2012 (Table S1). From late February to late July, TI (2012–2015) and AS (2016–2019) walked around the entire island using playback almost every night (from sundown to about midnight), except when it rained (see Sawada, Iwasaki, Inoue, et al., 2021). The coordinates of each encounter with territorial owls, along with identity and sex, were recorded. Individuals were identified by unique combinations of colored reflective tape wrapped around metal leg rings (Takagi, 2020) using binoculars from a distance of about 10 m.

### 2.4 Body measurements

Almost all breeding individuals (identified during breeding monitoring from 2002 onwards), and unmarked individuals (encountered during territory identification surveys from 2012 onwards) were captured by mist‐net, ringed and measured. The measurements of 778 individuals are used in this study. Body mass (to the nearest 0.1 g) was measured using a Pesola spring balance or a digital weighing scale. Tarsus length, culmen length, bill depth, bill width, head length, and tail length (to the nearest 0.01 mm) were measured with an electronic digital caliper. Wing length (to the nearest 0.5 mm) was measured with a stainless‐steel ruler. Measurements were made twice or more during each capture, allowing the use of mean values (see Table 1; Sawada, Iwasaki, Matsuo, et al., 2021). Since the correlations of these variables were weak, the values of the variables were used in the analysis as they were (Figure S1). For the analysis of size assortative mating, we used the measurements of individuals that were confirmed as present from 2012 onwards, because randomization tests (see below) require detailed territory data which has only been available since 2012. However, for the analysis of reproductive success we have used measurements of individuals from 2007 onwards. The owls were captured and handled under license from the Yamashina Institute for Ornithology and the Ministry of the Environment Japan (from No. 11‐64 and 11‐65 in 2003 to No. 11‐138 and 11‐140 in 2019).

**TABLE 1:** Definition of the morphometric traits measured in this study

Trait	Definition
Body mass	Body mass
Tarsus length	From the base to the tip of the tarsometatarsus
Culmen length	From the base to the tip of the bill
Bill depth	Height of the closed bill at the anterior end of nostril vertical to the gape
Bill width	Width of the bill at the anterior end of nostril
Head length	From the back of the skull to the tip of the bill
Tail length	From the root to the tip of the central rectrix
Wing length	From the bend of the wing to the tip of the longest primary at a flattened state

### 2.5 Sex and age determination

Sex was determined by vocal characteristics, by the presence of a brood patch, or by PCR amplification of the Chromo Helicase DNA‐binding gene (Fridolfsson & Ellegren, 1999; Sawada, Iwasaki, Matsuo, et al., 2021; Takagi, 2020). Age class (yearling or adult) was estimated from plumage characteristics following Sawada, Iwasaki, Matsuo, et al. (2021). Age, as used in the analyses below, refers to this dichotomous classification and not an exact age in years. In brief, if a bird meets two criteria out of three (pointed primaries, soft primary rachides, and worn secondaries), we judged the bird yearling. Detailed procedures for sexing and aging the owls are described in Sawada, Iwasaki, Matsuo, et al. (2021).

### 2.6 Statistical analysis

#### 2.6.1 Data standardization before analysis

Before analysis, measurement data were statistically controlled for differences between measurers and years (Grant & Grant, 2008; Green, 2019), using the results of generalized linear mixed models fitted to the dataset collected during the same period in Sawada, Iwasaki, Matsuo, et al. (2021; See Appendices 1 and 2, and Tables S2 and S3 for detailed standardization procedures). Data standardization and all analyses below were conducted using R 4.1.1 (R Core Team, 2021).

#### 2.6.2 Fundamental analysis of assortative mating

To describe size assortative mating, we calculated Pearson's correlation between measurements of mated males and females. By using the first measurements that were collected for each individual (some individuals were measured in multiple years), the effects of “become alike” were excluded as much as possible. The significance of the correlation was tested based on two methods, the *cor.test* function in R (hereafter, “parametric test”), and a randomization test (Erlandsson & Rolán‐Alvarez, 1998). A parametric test was conducted because it is the commonest method to document assortative mating. A randomization test was conducted because the assumptions of the parametric test can be violated in the data of assortative mating (i.e. non‐normality and/or non‐independence).

The procedures of the randomization test were similar to those described by Sawada et al. (2020): (step 1) Using a data matrix containing data for all territories in all years, males are randomly assigned to females within each year. Here, we randomly choose the same number of females as the actual pair data; (step 2) Calculate Pearson's correlation coefficient based on these simulated pairs; (step 3) Repeat processes in the step one and step two 1000 times; (step 4) Generate a distribution of correlation coefficients from these simulated values. This distribution is used as the null distribution of correlation coefficients expected under random mating in this owl population; (step 5) Obtain two tailed *p*‐values as twice the proportion of simulated values, which are more extreme than the actual values. For the traits for which we found significant assortative mating by both the parametric and randomization tests (culmen length and wing length, see Section 3), we further investigated the generating mechanisms of the assortment by the analyses detailed in the following sections.

#### 2.6.3 Mechanism 1: Like meets like

We took two approaches to test whether mechanism 1 (like meets like) contributes to the assortment; first, statistical control of spatial and temporal accessibility of potential mates in the randomization test, second, testing whether the spatially accessible individuals were similar‐sized or not and third temporally accessible individuals were similar‐sized or not.

The basic premise of the first approach is that non‐significance after controlling for mechanism 1 is indicative of contribution of mechanism 1 to the significance detected above (Erlandsson & Rolán‐Alvarez, 1998). We modified step1 of the previously described randomization test so as to consider the spatial or temporal accessibility to potential mates.

The median dispersal distance of females is 1145 m (Matsuo, unpublished data; Sawada et al., 2019) so, to control for spatial accessibility, we randomly assigned a male within that distance of the focal female to that female. Then, the null distribution and *p*‐value were calculated as the same way. If the distribution moves in the direction of the actual value of the correlation coefficient and the *p*‐value increases, then mating with spatially more accessible mates would explain the size assortment. In this owl population, males settle before females and females exhibit roaming dispersal behavior indicating female's assessment of males (Sawada & Takagi, unpublished data). Therefore, we consider that assignment of males to females reasonably mimics their pair formation process.

To control for temporal accessibility of potential mates, we randomly assigned males while considering the age of females and males. There are three reasons for this treatment: (1) There is a tendency for age assortative mating (see Appendix 3; Table 2); (2) Yearlings tend to breed late in a breeding season, probably due to their late pair formation (see Appendix 3; Tables S4 and S5); (3) Yearlings and adults differ slightly in size (Sawada, Iwasaki, Matsuo, et al., 2021).

**TABLE 2:** Result of Fisher's exact test for age assortative mating

Year	M_Y_F_Y_	M_Y_F_A_	M_A_F_Y_	M_A_F_A_	*N*	*p*	*p*_correct_
2012	0	1	0	21	22	1.000	1.000
2013	1	2	1	16	20	0.284	1.000
2014	2	6	0	11	19	0.164	0.982
2015	1	1	2	11	15	0.371	1.000
2016	7	10	5	12	34	0.721	1.000
2017	4	11	4	10	29	1.000	1.000
2018	7	8	5	27	47	0.034	0.239
2019	13	9	5	27	54	0.001	0.010
Total	35	48	22	135	240	0.000	0.000
Frequency	0.146	0.200	0.092	0.563			

Abbreviations: M_Y_: male yearling, M_A_: male adult, F_Y_: female yearling, F_A_: female adult, MF: number of pairs of the age combination denoted by subscripts, *N*: number of pairs in total, *p*: *p*‐Values of a Fisher's exact test for age assortative mating (Appendix 3), *p*_ccorect_: *p*‐Values adjusted by Holm's method.

Let *P*_YY_, *P*_YA_, *P*_AY_, and *P*_AA_ be the observed frequency of pairs (first and second subscripts denote age (yearling or adult), of males and females, respectively). To mimic the pair formation based on age, for yearling females, yearling males were assigned with the probability *P*_YY_ and adult males were assigned with the probability *P*_AY_. For adult females, yearling males were assigned with the probability *P*_YA_ and adult males were assigned with the probability *P*_AA_. Then, null distribution and *p*‐value were calculated as the same way. If the distribution moves in the direction of the actual value of the correlation coefficient and the *p*‐value increases, then mating with temporally more accessible, similar‐aged mates, would explain the size assortative mating. We used the means of the observed frequencies of pairs from 2012 to 2019 as the values of *P*_YY_, *P*_YA_, *P*_AY_, and *P*_AA_ (Table 2).

To assess similarity among spatially accessible individuals, we described and tested spatial autocorrelation in body size by Mantel test (Mantel, 1967; Appendix 4). We focused on geographic distance and size difference between females and males because our interest is in whether spatially accessible males for females are similar to the females. This test was applied to all years (2012–2019) separately for culmen length and wing length. Holm's correction of *p*‐value was applied to each trait.

We also investigated the heritability of culmen length and wing length. This was motivated by the fact that previous research has suggested that there is a spatially autocorrelated genetic structure (Sawada et al., 2019), and that if heritable components of body size variation exist, this may translate into the spatial heterogeneity in morphological variation. To estimate heritability, we applied parent–offspring regression to father–mother–offspring triads identified during breeding monitoring and territory mapping surveys (63 triads for culmen length and 61 triads for wing length). Regression coefficients in the regressions of offspring values over hypothetical single intermediate parents (midparent) values were used as an estimate of heritability (Lynch & Walsh, 1998).

To assess similarity among temporally accessible individuals, we described size difference between individuals which settled at age class of yearling (early‐settlers) and individuals which settled at age class of adult (late‐settlers). Since we do not accurately know their breeding status (although they breed in most case), we refer to them “settlers,” not “breeders.” If both male early‐settlers and female early‐settlers have similar body size, similar sized individuals are likely to meet. Based on these considerations, culmen length and wing length of early‐settlers (46 males and 20 females) and late‐settlers (12 males and 17 females) were compared by *t*‐test in each sex. Data were obtained from territory identification.

#### 2.6.4 Mechanism 2: Become alike

We took two approaches to test whether mechanism 2 (become alike) contributes to assortment. First we compared the difference in body size of paired individuals when first measured and when last measured. The latter measurement is expected to reflect any changes in body size accumulated after pair formation. If mechanism 2 works, then the difference between mates when last measured is expected to be smaller than when first measured. Furthermore, such change might be more pronounced in a labile trait such as wing length than in a less variable bony trait such as culmen length. We tested these expectations by paired *t*‐test.

Second we compared correlation coefficients calculated from first measurements with those from last measurements. The statistical significance of the difference between the two correlation coefficients was tested using Fisher's *Z* transformation of correlation coefficients (see Appendix 5; Zou, 2007), with a strong positive correlation in last measurements suggesting that mechanism two does contribution to assortative mating in the owls.

On the other hand, there are some limitations in these approaches detecting the effect of “become alike.” First the analyses do not account for body size change due to growth and senescence. If body size shows bell‐shaped change along their lifetime (e.g. body mass may increase at their young period but decrease in their old period), taking difference of just two measurements may not be able to detect precise pattern of “become alike.” Second the analyses do not account for time span between the first measurement and the last measurement. Again, if the body size shows bell‐shaped change along their lifetime, when the measurements were taken is important. Without the information, the analyses may miss the evidence of “become alike.” Nevertheless, it is difficult to deal with these problems in our dataset, since exact age is unknown for most individuals. Therefore, it should be noted that analyses for “become alike” are conservative in this study.

#### 2.6.5 Mechanism 3: Mate choice

We took three approaches to test whether mechanism 3 (mate choice) contributes to assortment. The first approach involved the statistical control of other mechanisms in the fundamental correlational analysis described above, based on the premise that persistent significant correlation, after controlling for other mechanisms, is indicative of contribution by mechanism 3 (Erlandsson & Rolán‐Alvarez, 1998). Because correlation analysis using first measurement data already minimizes the effect of mechanism 2 (become alike), we considered to controlling for mechanism 1 (like meets like). The detailed procedures are the same as those described above, for testing mechanism 1.

The second approach consisted of an analysis of fitness components. The premise behind this is that, if there is active mate choice with respect to body size, then choosers are likely to benefit from this behavior (Andersson & Simmons, 2006). To test this, we evaluated the effects of body size on the number of fledglings reared at single breeding attempt, on the number of recruits at single breeding attempt, and on the survival rate. Because pairs breed together over successive years, a small increment in reproductive success during a single breeding attempt can be magnified when focusing on lifetime reproductive success. Because longevity positively correlates with lifetime reproductive success in this owl population (Sawada et al., 2020), increased survival rate indicates increased fitness. Generalized linear models (GLM) and GLMM with log‐link and Poisson distribution by *glm* and *glmer* function in lme4 package (Bates et al., 2015) were used for the analysis of fledglings and recruits. Bayesian implementation of the Cormack‐Jolly‐Seber model (CJS model, Stan Development Team, 2018) was used for the analysis of survival rate. The essence of the analyses is described below, and details are given in Appendices 6 and 7 and Table S6.

For the number of fledglings and recruits, we constructed models with all possible combinations of fixed effects (Year, Egg laying date, Father age, Mother age, Father culmen length, Father wing length, Mother culmen length, Mother wing length, Difference in culmen length between parents, Difference in wing length between parents), and all possible combinations of random effects (Mother ID, Father ID). Then, we searched for the best combination of them in terms of AIC using the *dredge* function in the MuMIn package (Barton, 2019). Top‐ranked models with ΔAIC <2 (difference from minimum AIC smaller than 2) were model‐averaged by *model.avg* to obtain model‐averaged regression coefficients and corresponding *p*‐values. Results were interpreted from the top model and the averaged model.

For the survival rate, we constructed a CJS model with survival rate modeled by five fixed effects (Year, Sex, Age, Culmen length, Wing length) and detection probability modeled by two fixed effects (Sex, Researcher; Sawada, Iwasaki, Inoue, et al., 2021). To consider sex‐dependency of the effect of body size, regression coefficients for body size (Culmen length, Wing length) were modeled to be sex‐dependent. These effects were introduced by GLM with logit link and Bernoulli distribution. Model implementation was done using Stan (Carpenter et al., 2017) and RStan (Stan Development Team, 2019). Fitting parameters were as follows: warmup = 15,000; iter = 5000; thin = 4. Convergence was confirmed based on Rhat diagnostic statistics and the output of *check_hmc_diagnostics* function in the rstan package. Significance of the fixed effects was judged based on whether the 95% credible intervals (95% CRI) include zero or not.

The third approach was a comparison of body size between female early‐settlers and female late‐settlers. The premise behind this analysis is selection of males by females. As already mentioned, in this population, females show roaming dispersal pattern and settle in territories which are held by males. In addition, there are more males than females (Sawada, Iwasaki, Inoue, et al., 2021). For males, rejecting females visiting their territories may not be a good choice. Therefore, female choice seems to have importance in this population. Then, females that disperse and settle earlier may have more potential mates to choose. If there are specific characteristics among early female settlers, then the advantage of specific females in acquiring specific males is suggested. Analysis is the same *t*‐test used to assess similarity among temporally accessible individuals in the tests for “like meets like.”

## 3 RESULTS

### 3.1 Fundamental analysis of assortative mating

**FIGURE 1:**
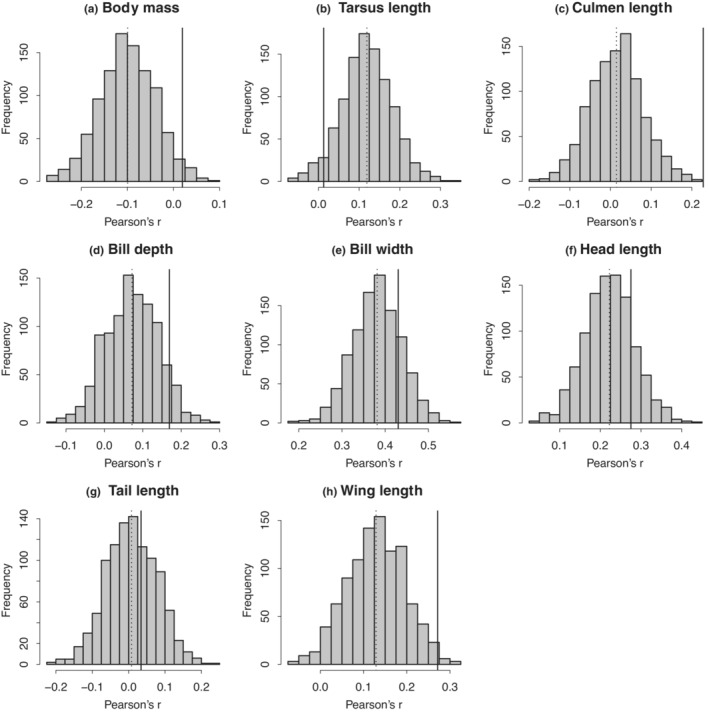
Results of randomization tests without any statistical controls. Histograms: Distribution of simulated correlation coefficients. Solid lines: Observed values. Dotted lines: means of histograms.

Parametric tests of correlations of body size measurements revealed that there was significant assortative mating with regards to culmen length, bill depth, bill width, head length, and wing length (Table 3; Figure S2). Significant assortments in all traits remained after *p*‐value corrections (Table 3). Randomization tests revealed significant assortative mating with regards to culmen length and wing length (Table 3; Figure 1). The assortment in culmen length even remained after *p*‐value corrections (Table 3). Because null distributions generated by randomization of all traits, except culmen length and tail length, did not have means near zero (Table 3; Figure 1), the significance of the parametric tests seemed to be overestimated. In subsequent analyses for generating mechanisms of assortment, we focused on culmen length and wing length as both tests identified significant assortment for these characteristics.

**TABLE 3:** Results of fundamental correlation analyses

Trait	*N*	*r*	*P*_param_	*P*_param_^§^	*P*_rand_	*P*_rand_^§^	*r*_last_	*P*_param_last_	*P*_param_last_^§^	*Z*	*P*_diff_
Body mass	240	0.019	0.768	1.000	0.056	0.336	0.004	0.954	1.000	0.168	0.867
Tarsus length	237	0.013	0.844	1.000	0.116	0.580	0.034	0.607	1.000	−0.225	0.822
Culmen length	236	0.228	**0.000**	**0.002**	**0.000**	**0.000**	0.131	**0.044**	0.222	1.083	0.279
Bill depth	236	0.169	**0.009**	**0.037**	0.148	0.592	−0.006	0.931	1.000	1.906	0.057
Bill width	236	0.430	**0.000**	**0.000**	0.370	1.000	0.430	**0.000**	**0.000**	0.006	0.995
Head length	236	0.275	**0.000**	**0.000**	0.366	1.000	0.365	**0.000**	**0.000**	−1.076	0.282
Tail length	213	0.034	0.620	1.000	0.702	1.000	0.067	0.330	1.000	−0.338	0.736
Wing length	230	0.271	**0.000**	**0.000**	**0.026**	0.182	0.208	**0.001**	**0.009**	0.712	0.477

*Note*: *N*: number of pairs, *r*: correlation coefficient calculated from first‐time measurements, *r*_last_: correlation coefficient calculated from last‐time measurements, *p*_param_: *p*‐value of parametric test on first‐time measurements, *p*_rand_: *p*‐value of randomization test on first‐time measurements, *p*_param_last_: *p*‐value of parametric test on last‐time measurements, §: Values after control of multiple testing by Holm's correction, *Z*: test statistic of difference between *r* and *r*_last_, *p*_diff_: *p*‐value corresponding to *Z*, Bold: *p*‐value <.05.

### 3.2 Test of mechanism 1: Like meets like

**FIGURE 2:**
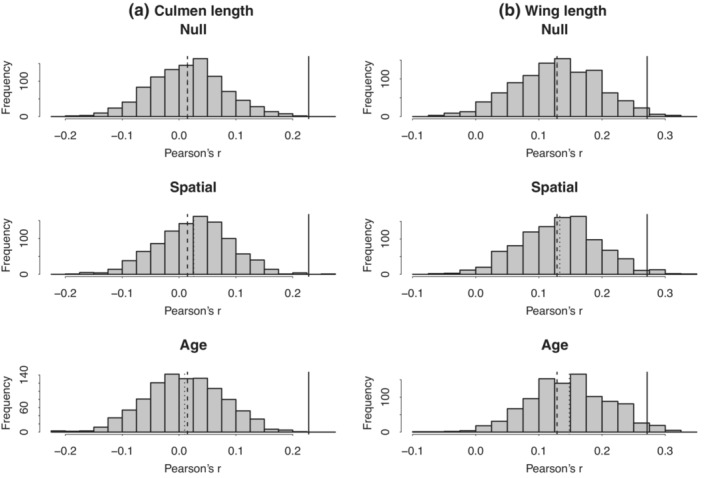
Results of statistical control of “like meets like” mechanisms in randomization tests for (a) culmen length and (b) wing length. Histograms: Distribution of simulated correlation coefficients. First row (null): Simulation without any controls, second row (spatial): Simulation with control for spatial accessibility, third row (age): Simulation with control for age‐assortment. Solid lines: Observed values. Dotted lines: Means of histograms. Dashed lines: Means of histograms of simulation without any controls in the first row.

Statistical control of spatial accessibility of potential mates hardly moved the null distribution toward actual values (Figure 2). *p*‐values were almost unchanged (Table S7, from *p* < .001 to .002 in culmen length, from *p* = .026 to .034 in wing length).

Statistical control of the age of potential mates hardly moved the null distribution toward actual values (Figure 2). *p*‐values were almost unchanged (Table S7, from *p* < .001 to .001 in culmen length, from *p* = .026 to .050 in wing length).

Heritability was significant for culmen length (Figure S3, h^2^ ± SE = 0.303 ± 0.119, *t* = 2.552, df = 61, *p* = .013) and for wing length (Figure S4, h^2^ ± SE = 0.245 ± 0.109, *t* = 2.252, df = 59, *p* = .028). No significant morphological similarity between females and their spatially accessible potential mates was found (Mantel test, all *p* > .05, Table S8).

**FIGURE 3:**
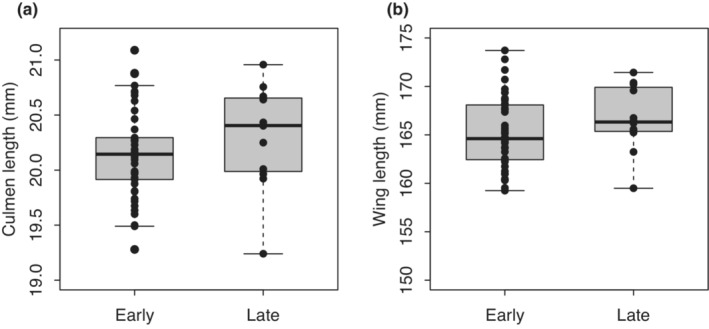
Comparison of early‐ and late‐settling males. (a) Culmen length, (b) wing length. Black dots: Data points.

**FIGURE 4:**
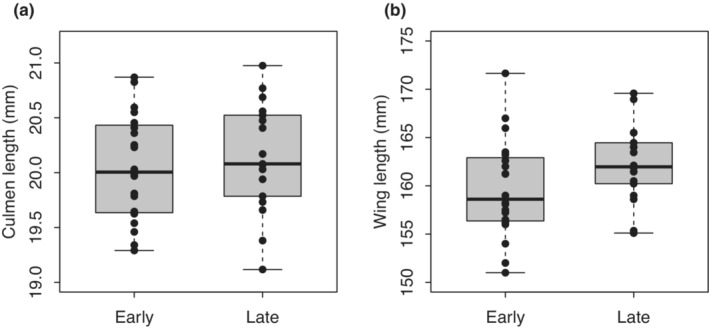
Comparison of early‐ and late‐settling females. (a) Culmen length, (b) wing length. Black dots: Data points.

When comparing early‐settlers and late‐settlers, culmen length did not differ significantly in males (Figure 3a, Welch's *t*‐test, *t* = −1.056, df = 15.486, *p* = .307) and females (Figure 4a, Welch's *t*‐test, *t* = −0.538, df = 33.494, *p* = .595). Wing length also did not differ significantly in males (Figure 3b, Welch's *t*‐test, *t* = −1.311, df = 17.970, *p* = .206). However, early‐settling females had slightly shorter wings than later‐settlers, although the difference was statistically marginal (Figure 4b, Welch's *t*‐test, *t* = −1.664, df = 34.810, *p* = .105).

### 3.3 Test of mechanism 2: Become alike

**FIGURE 5:**
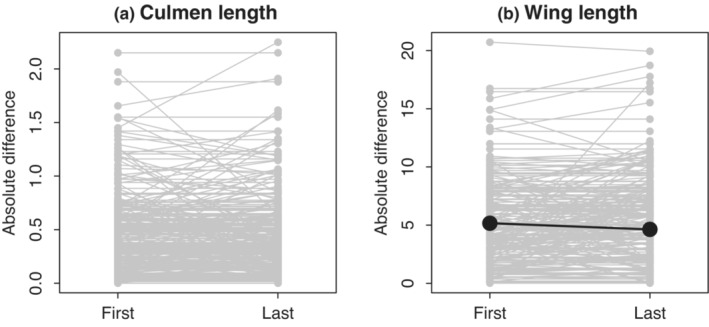
Comparison of body size difference of paired individuals when first measured and when last measured. (a) Culmen length, (b) wing length. Gray dots and gray lines: Each pair. Black dot and black line: Mean estimate (shown when paired *t*‐test detected significant difference).

For culmen length, differences in last measurements and first measurements did not differ significantly (Figure 5a, paired *t*‐test, *t* = 0.354, df = 235, *p* = .724). However, for wing length, the difference in last measurements was significantly smaller than the difference in first‐time measurements (Figure 5b, paired *t*‐test, *t* = −3.192, df = 229, *p* = .002).

Correlation coefficients calculated from last measurements (Figure S4) were not significantly different from the coefficients calculated from first measurements (Table 3, all *p* > .05). However, the correlation coefficients of bill depth marginally weakened from 0.169 to −0.006 (Table 3, *Z* = 1.906, *p* = .057).

### 3.4 Test of mechanism 3: Mate choice

Statistical control of spatial and temporal accessibility of potential mates did not completely cancel the significance of the correlation coefficients for culmen length and wing length, as described above (Figure 2; Table S7).

**FIGURE 6:**
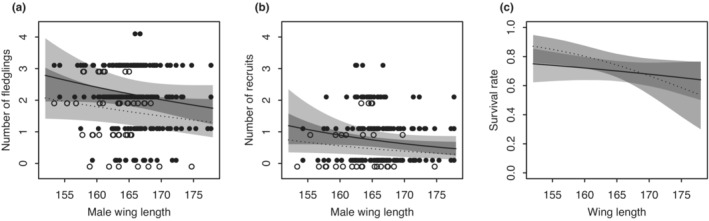
Relationships between wing length and fitness components. (a) Wing length of males and number of fledglings they produced at one breeding attempt. (b) Wing length of males and number of recruits they produced at one breeding attempt. Black dots and solid lines: Data points and mean estimates of adult males, white dots and dotted lines: Data points and mean estimates of yearling males. (c) Wing length and survival rate. Solid line: Males, dotted line: Females. Pale gray area: 95% CRI of mean estimates, dark gray area: Overlap of the 95% CRI.

The best model for number of fledglings was one that included year, male age, and male wing length (Table S9). From this model, the number of fledglings of pairs involving yearling males was 0.74 times lower than pairs involving adult males (Figure 6a; Table S10, coefficient ± SE = −0.30 ± 0.13, *Z* = −2.24, *p* = .03), and a 10 mm reduction in male wing length increased the number of fledglings 1.20 times (Figure 6a; Table S10, coefficient + SE = −0.02 ± 0.01, *Z* = −1.56, *p* = .12) although this was not significant. Applying model averaging to the best set of models, effect size and significance of male age and male wing length were almost unchanged (Table S11).

The best model for number of recruits was a model including year, egg laying date, male age, and male wing length (Table S12). From this model, the number of recruits in pairs involving yearling males was 0.62 times lower than pairs with adult males (Figure 6b; Table S13, coefficient ± SE = −0.47 ± 0.28, *Z* = −1.67, *p* = .10); 10 days late egg laying reduced the number of recruits by 0.85 times (Table S13, coefficient ± SE = −0.02 ± 0.01, *Z* = −1.63, *p* = .10), and a 10 mm reduction in male wing length increased the number of recruits 1.44 times (Figure 6b; Table S13, coefficient ± SE = −0.04 ± 0.02, *Z* = −1.68, *p* = .09), although the effects were marginal. Applying model averaging to the best set of models, effect size and significance of male age and male wing length were almost unchanged (Table S14).

Survival rate was not significantly affected by culmen length in either sex (Figure 6c; Table S15, coefficient for male = 0.03, 95% CRI = [−0.14, 0.20]; coefficient for female = −0.16, 95% CRI = [−0.39, 0.07]). However, it was significantly affected by wing length of females though not of males (Figure 6c; Table S15, coefficient for female = −0.32, 95% CRI = [−0.63, −0.03], coefficient for male = −0.10, 95% CRI = [−0.28, 0.09]). Age had a significant effect (Table S15, mean = 4.79, 95% CRI = [2.35, 9.54]).

Early‐settling and late‐settling females did not differ significantly in culmen length and wing length, although early‐settlers had slightly shorter wings than later‐settlers in females as described above (Figures 3 and 4).

## 4 DISCUSSION

In this study of size assortative mating of the Ryukyu Scops Owls on Minami‐daito Island, we found significant assortative mating for culmen length and wing length by two statistical approaches (parametric and randomization tests). Statistical control of spatial and temporal accessibility in the test did not cancel the assortment. Females settling in their first year tend to have small wings, and males with short wings tend to have good fitness components. The differences in wing lengths of paired individuals were smaller later in their paired period than in their early paired period. We discuss possible generating mechanisms of assortative mating in this owl.

### 4.1 Possible mechanism of assortative mating

For both culmen length and wing length, active mate choice (mechanism 3) is a likely explanation for the assortative mating in this owl population. There are three reasons for this. First, statistical control of spatial temporal accessibility did not cancel the assortment. Second, Mantel test did not find significant similarity between females and potential mates. Third, early settlers and late settlers did not significantly differ in culmen length in both sexes. These three results indicate little contribution of mechanism 1 “like meets like” to the assortment. Fourth, our results already minimize the effect of mechanism 2 “become alike” by using first measurement data.

For the assortment with respect to culmen length, there was no further support of the interpretation above, because no fitness components correlated with the trait. However, for the assortment with respect to wing length, mate choice is further supported for two reasons. First, males with short wings have an advantage in reproduction, which is suggested by the fact that males with shorter wings produced slightly more fledglings and recruits in a single breeding attempt (albeit not significantly). Females would benefit from choosing short‐winged males. Considering that pair‐bond lasts successive years, any small increment in reproductive success during a single breeding attempt may be magnified over their lifetimes. Second, females with short wings have an advantage when mating with males with short wings, which is suggested by the fact that females which settled in their first year had slightly (but not significantly) shorter wings. Short‐winged females are likely to have advantages in acquiring territories or mates, indicating that short‐winged females have priority in accessing males with good reproductive performance.

Nevertheless, the contribution of mechanism 2 “become alike” cannot be completely ruled out for the assortment by wing length because of the small size difference after late pair formation. We used measurements made when we first captured individuals to replicate as closely as possible any correlation at the time of pair formation. However, we were unable to obtain measurements at the exact timing of pair formation. Thus we are faced by the limitation that, if the wing lengths of mated individuals become alike after pair formation, our first measurements may already have been after the mechanism “become alike” began operating, generating a positive correlation between males and females. However, because the Mantel test for spatial autocorrelation structure did not detect similarity in wing length between nearby individuals, sharing similar habitat may not lead to similarity between individuals indicating that “becoming alike” is unlikely.

Physical constraint (Crespi, 1989), a mechanism that we did not address in this study, also may not be ruled out. For some species of arthropod with long copulation time and or specific copulatory behavior, inefficiency in copulation between mates with a large size difference is suggested as a cause of size assortment (Han et al., 2010). Compared with arthropods that may remain in contact for several hours (Crespi, 1989), the duration of avian copulation is very much shorter, lasting for only a few seconds, or at most several tens of seconds (Birkhead et al., 1987). Whether the physical constraint is important or unimportant in brief copulation by birds are unknown at present.

### 4.2 Costs and benefits of body size

If short‐winged males have good fitness components and short‐winged females can settle early, what costs and benefits produce this tendency? For males, short wings would have benefits in flying and hunting ability (Hakkarainen et al., 1996; Mueller, 1986) and have costs in physical fighting (McDonald et al., 2005). However, the owls rely on vocal contest in territory competitions and physical competitions are rarely observed (Bai & Severinghaus, 2012). Therefore, benefits of short wings may outweigh costs for males. For females, short wings would have benefits in hunting efficiency, again (Massemin et al., 2000) and have costs in breeding behaviors such as egg production and incubation (Krüger, 2005; Mueller, 1986). Importantly, these costs at breeding matter after settling, whereas the benefits of hunting efficiency matters even before settling. Therefore, at least before settling, benefits of short wings may outweigh costs also for females.

### 4.3 Mechanisms to assess body size

A question we did not address in this study is, how do the owls know the body size of other individuals? Because of their nocturnality, owls rely heavily on vocal communication (König & Weick, 2008), and may perhaps use vocal characteristics to infer body size. In this owl, body size (tarsus length, culmen length, bill width, head length, tail length, body mass) correlates with hoot frequency (Takagi, unpublished data). Recording the behavioral responses to hoots at various frequencies would be a promising way to answer this problem in future research (Podos, 2010). Nevertheless, body size may also be assessed visually, and plumage characteristics may also be important (Galeotti & Rubolini, 2007).

### 4.4 Taxonomic differences in the generating mechanisms of assortative mating

The relative contribution of previously proposed mechanisms for assortative mating may depend on taxonomic group. For arthropods and fishes, large males have an important advantage in competition for large fecund females (Crespi, 1989; de Almeida Borghezan et al., 2019; Taborsky et al., 2009). This implicitly assumes that female fecundity (number of eggs) increases with body size. However, some taxonomic groups, such as birds, do not conform to this assumption since their females produce far fewer eggs than either arthropods or fishes. Therefore, mate choice mechanisms that are not based on female fecundity may be more important, because the merits of competing for large females seems to be limited. Support for the “like meets like” mechanism actually exists (Hedenström, 1987; Indykiewicz et al., 2017). However, “mate choice” cannot be ignored. Catry et al. (1999) did a rare study into the causes of size assortative mating in birds (skuas and jaegers) which exhibit reversed sexual size dimorphism. They suggested that small males had an advantage in acquiring mates because females rejected large males. Therefore, male smallness, rather than female largeness, may be important for assortative mating in species with reversed sexual dimorphism. Similarly, our study supports a mechanism whereby small females have an advantage in acquiring small males with good reproductive performance. This is a simple corollary from the traditional explanation of size assortative mating in which large males have an advantage in acquiring large females with good fecundity.

## 5 CONCLUSION

We have shown that size assortative mating, with respect to culmen length and wing length, occurs in the Ryukyu Scops Owl, and that mate choice is a possible mechanism contributing to the assortment. Specifically, small females seemed to choose small males which are expected to give good reproductive outputs for the females. The background of that choice may be the benefit of being small in terms of flying and hunting ability. Since reports of size assortative mating in raptor species often only describe whether it occurs, future studies should focus on the causes of the assortment. Our understanding of size assortative mating has been constructed mainly based on organisms with non‐reversed sexual size dimorphism, thus focusing also on those with reversed sexual size dimorphism will contribute to extending our understanding.

## AUTHOR CONTRIBUTIONS

**Akira Sawada:** Conceptualization (equal); data curation (lead); formal analysis (lead); funding acquisition (equal); investigation (equal); methodology (equal); project administration (equal); resources (equal); supervision (supporting); validation (equal); visualization (lead); writing – original draft (lead); writing – review and editing (lead). **Tetsuya Iwasaki:** Conceptualization (equal); data curation (supporting); investigation (equal); methodology (equal); project administration (equal); resources (equal); supervision (supporting); validation (supporting); visualization (supporting); writing – review and editing (supporting). **Kana Akatani:** Data curation (equal); investigation (equal); methodology (equal); project administration (equal); resources (equal); supervision (supporting); writing – review and editing (supporting). **Masaoki Takagi:** Conceptualization (equal); formal analysis (supporting); funding acquisition (lead); investigation (equal); methodology (equal); project administration (lead); resources (equal); supervision (lead); validation (equal); visualization (supporting); writing – original draft (supporting); writing – review and editing (supporting).

## FUNDING INFORMATION

This study was funded from Pro Nature Foundation Japan, Inui Memorial Trust for Research on Animal Science, The Zoshinkai Fund For Protection of Endangered Animals, Sasakawa Scientific Research Grant from the Japan Science Society, Japan Bird Research Association, Suntory Fund for Bird Conservation, Seven‐Eleven Midori no Kikin, Mont‐bell, JSPS Kakenhi (Grant Number 17770019, 21570022, 16H04737, 19J12833 and 21J00958) and Tokyo Zoological Park Society.

## CONFLICT OF INTEREST

The authors declares that no conflicts of interest exist.

## Supporting information


Figure S1

Figure S2

Figure S3

Figure S4

Figure S5

Figure S6

Figure S7

Tables S1‐S15

Appendix S1

## Data Availability

All data will be archived at Dryad upon acceptance.

## APPENDIX 1

### STANDARDIZATION PROCEDURES

Before analysis, all measurement data were statistically controlled for differences between measurers and years to avoid false positive correlation which can occur by pooling heterogeneous measurement data. Using Sawada, Iwasaki, Matsuo, et al.'s (2021) estimates of the effect of measurers and years, all measurements were standardized to the measurement in 2019 by AS. For example, as culmen length measurements by TI and AS were estimated to be −0.04 and 0.21 mm larger than by KA, 0.21 − (−0.04) = 0.25 mm was added to measurements by TI so that the value obtained by him to take the same mean as the measurements by AS (Figure S5). Effect of year was adjusted in the same manner. Values used for this adjustment are given in Tables S2 and S3 of this study and also in the supplementary tables in Sawada, Iwasaki, Matsuo, et al. (2021). If such the standardizations are not applied, correlation coefficients calculated from the raw data become inflated. Reason why such inflation occurs is explained by Figure S6. Before the standardizations, data scatter due to effect of measurer. Therefore, if we calculate correlation coefficient ignoring the effect of measurer, absolute value of the coefficient becomes large due to the long‐stretched distribution (Figure S6a). However, such the effect can be controlled for by standardizing the effect of measurer a priori (Figure S6b).

## APPENDIX 2

### DATA MATRIX

Combining the measurement data and data from breeding monitoring, territory mapping, and sex and age determination, five data frames (A–E) were generated on R so that the subsequent analyses could immediately use the data (Figure S7).

Data matrix A contains the data for all territories of all years, with each row corresponding to a territory in a year. It contains UTM coordinates, the identities of the territory holders (males and females), ages of the territory holders, and body measurements of the territory holders in the year. Because not all individuals were measured in all years when they were present, means of measurements were used as surrogate measurements in such data‐lacking year. These data were used for correlation analysis by randomization test, analysis of similarity of body size between females and their potential mates by Mantel test, and used for generating data matrices B, D, and E.

Data matrix B is derived from matrix A. Matrix B contains rows of matrix A that correspond to territories where both male and female territory holders were ringed. Duplicated pairs in matrix A are also omitted from matrix B, except in the year when they were first identified as a pair. Each row contains UTM coordinates, the identities of the territory holders (males and females), ages of the territory holders, first and last measurement data for corresponding individuals if they were measured across multiple years. These data were used for correlation analysis by parametric test, correlation analysis by randomization test, and analyses for mechanism 3.

Data matrix C contains the data for all breeding attempts, with each row corresponding to a breeding attempt. Matrix C contains, egg laying date, the identities of the father and mother, ages of the parents, and their body measurements. Unlike matrix A, means across years were used as measurement data in this matrix, because it was used for analysis of relationships between reproductive success and attributes of parents. These data were used for the analyses of number of fledglings, number of recruits and egg laying date, and used for generating data matrix E.

Data matrix D contains survival history, with the number of rows equal to the number of individuals marked or recaptured (resighted) from 2012 to 2019. The eight columns represent the number of years in which we marked or recaptured (resighted) the individuals (= 2019–2012 + 1). Each row corresponds to an individual. If an individual *i* was captured or recaptured (resighted) in year 2011 + *j*, the (*i*, *j*) element of the matrix is 1, otherwise it was 0. This matrix was generated based on matrix A. These data were used for survival analyses using the Cormack‐Jolly‐Seber model (CJS model) and analyses of size difference between early settlers and late‐settlers.

Data matrix E contains the measurement data for father–mother–offspring triads. As these data were used for parent–offspring regression, and therefore there should be one measurement value for each individual, mean values across years were used for measurement data in the matrix. These data were used for analyses of parent–offspring regression.

## APPENDIX 3

### CONTROLLING FOR TEMPORAL ACCESSIBILITY

To control for temporal accessibility of potential mates in randomization tests, we randomly assigned males while considering the age of females and males. There are three reasons for this treatment: (1) There was a tendency for age assortative mating (see below); (2) Yearlings tend to breed late, probably due to their late pair formation (see below); (3) There is a slight size difference between yearlings and adults (Sawada, Iwasaki, Matsuo, et al., 2021).

To confirm age assortative mating, we applied Fisher's exact test on a cross table which contains frequencies of pairs of (1) yearling male and yearling female, (2) yearling male and adult female, (3) adult male and yearling female, and (4) adult male and adult female. The test was conducted for yearly data and for pooled data.

There were significant age‐assortative matings in some years (Table 2, Fisher's exact test, 2018, *p* = 0.034; 2019, *p* = 0.001) and throughout the study period (Table 2, Fisher's exact test, *p* = 0.000).

To show the late breeding of yearlings using data matrix C, we evaluated the effects of age on egg laying date. We constructed Normal GLMM models with all fixed effects (Year, Father age, Mother age), and all possible combinations of random effects (Mother ID, Father ID). Each egg laying date was standardized by subtracting the mean egg laying date of the year in which the data were collected.

We included these random effects because our data contained multiple data from the same parents. Varying the combination of random effects, a model with Mother ID gave the smallest AIC value (Table S4). Therefore, we used GLMM models with Mother ID in the subsequent analyses.

Then, we searched for best combination of fixed effects in terms of AIC using the dredge function in the MuMIn package. Because the best model gave an AIC value which was much smaller than the second model (ΔAIC = 6.17, Table S4), we interpreted the results without considering model averaging.

The best model for egg laying date included significant effects of male age and female age (Table S4). From this model, egg laying date in pairs with yearling males was 2.97 days later than pairs with adult males (Table S5, coefficient ± SE = 2.97 ± 1.31, *t* = 2.27, df = 257.24, *p* = 0.02) and egg laying date in pairs with yearling females was 4.79 days later than pairs with adult females (Table S5, coefficient ± SE = 4.79 ± 1.53, *t* = 3.13, df = 274.23, *p* = 0.00).

## APPENDIX 4

### MANTEL TEST

To assess similarity among spatially accessible individuals, we described and tested spatial autocorrelation in body size by Mantel test. We focused on geographic distance and size difference from females to males because our interest is whether spatially accessible males for females are similar to the females. Therefore, input matrixes were non‐square *n*_f_ × *n*_m_ matrix X and Y, where *n*_f_ and *n*_m_ are the number of females and males, respectively. Elements of the matrixes *x*_*ij*_ and *y*_*ij*_ are the geographic distance and absolute size difference between the corresponding female *i* and male *j*. Note that this is not a common setting for Mantel test because the test is often applied to a square matrix to ask, for example, whether “males” are similar to spatially neighboring “males.” Here, our question is whether “females” are similar to spatially neighboring “males.” We followed the original description of the test (Mantel, 1967) to apply the test to non‐square matrixes. Test statistic *M* was defined as ∑∑*x*_*ij*_ *y*_*ij*_ (summation is taken over all *i* and *j*). Null distribution of M and corresponding *p*‐value were obtained by permutation test (1000 permutations). This test was applied to all years (2012–2019) separately for culmen length and for wing length.

## APPENDIX 5

### TEST OF DIFFERENCE BETWEEN TWO CORRELATION COEFFICIENTS

We compared correlation coefficients at first and last measurements. Statistical significance was based on *Z* test using Fisher's *Z* transformation of correlation coefficients.

Let *z*_before_ and *z*_after_ be Fisher's *Z* transformation of correlation coefficients which are calculated from the first and last measurements, respectively. Test statistic *Z* and *p*‐value were calculated by the following formula:

$$Z=\frac{z_{\text{after}}-z_{\text{before}}}{\sqrt{\frac{1}{n_{\text{after}}-3}+\frac{1}{n_{\text{before}}-3}}}=\frac{z_{\text{after}}-z_{\text{before}}}{\sqrt{\frac{2}{n-3}}}\sim \text{Normal}\left(0,1\right)$$

Here, *n*_after_ and *n*_before_ are the sample size. In this study they are identical and written as *n*.

## APPENDIX 6

### NUMBER OF FLEDGLINGS

Using data matrix C, we evaluated the effects of body size on number of fledglings. We constructed Poisson GLMM models with all fixed effects (Year, Egg laying date, Father age, Mother age, Father culmen length, Father wing length, Mother culmen length, Mother wing length, Difference in culmen length between parents, Difference in wing length between parents), and all possible combinations of random effects (Mother ID, Father ID). Egg laying date is standardized by subtracting the mean egg laying date of the year.

We included the random effects because our data contained multiple data from the same parents. However, variance estimates in relation to the random effects were zero and, the model without the random effect gave a smaller AIC value than the model with the random effects (Table S6). Therefore, we dropped the random effects from subsequent analyses and used GLM instead of GLMM.

Then, we searched for the best combination of fixed effects in terms of AIC using the *dredge* function in the MuMIn package. Top‐ranked models with ΔAIC <2 (difference from minimum AIC smaller than 2) were model averaged by *model.avg* to obtain model averaged regression coefficients and corresponding *p*‐values. Specifically,“subset” coefficients in the output of *model.avg* were used for interpretation.

## APPENDIX 7

### NUMBER OF RECRUITS

Using data matrix C, we evaluated the effects of body size on the number of recruits. We constructed Poisson GLMM models with all fixed effects (Year, Egg laying date, Father age, Mother age, Father culmen length, Father wing length, Mother culmen length, Mother wing length, Difference in culmen length between parents, Difference in wing length between parents), and all possible combinations of random effects (Mother ID, Father ID). Egg laying date is standardized by subtracting the mean egg laying date of the year.

We included these random effects because our data contained multiple data from the same parents. However, variance estimates in relation to the random effects were zero, and the model without the random effects gave a smaller AIC value than the model with the random effect (Table S6). Therefore, we dropped the random effects from subsequent analyses and used GLM instead of GLMM.

Then, we searched for the best combination of fixed effects in terms of AIC using the *dredge* function in the MuMIn package. Top‐ranked models with ΔAIC <2 (difference from minimum AIC smaller than 2) were model averaged by *model.avg* to obtain model averaged regression coefficients and corresponding *p*‐values. Specifically, “subset” coefficients in the output of *model.avg* were used for interpretation.

## References

[ece39578-bib-0001] Akatani, K. (2011). Ryukyu Scops Owl Ryukyu‐konohazuku (Jpn) *Otus elegans* . Bird Research News, 8, 4–5.

[ece39578-bib-0002] Akatani, K. , Matsuo, T. , & Takagi, M. (2011). Breeding ecology and habitat use of the Daito Scops Owl (*Otus elegans interpositus*) on an oceanic island. Journal of Raptor Research, 45, 315–323.

[ece39578-bib-0003] Andersson, M. , & Simmons, L. W. (2006). Sexual selection and mate choice. Trends in Ecology & Evolution, 21, 296–302. 16769428 10.1016/j.tree.2006.03.015

[ece39578-bib-0004] Bai, M. L. , & Severinghaus, L. L. (2012). Disentangling site and mate fidelity in a monogamous population under strong nest site competition. Animal Behaviour, 84, 251–259.

[ece39578-bib-0005] Barton, K. (2019). MuMIn: Multi‐Model Inference, Version 1.43.6.

[ece39578-bib-0006] Bates, D. , Mächler, M. , Bolker, B. , & Walker, S. (2015). Fitting linear mixed‐effects models using lme4. Journal of Statistical Software, 67, 1–48.

[ece39578-bib-0007] Birkhead, T. R. , Atkin, L. , & Møller, A. P. (1987). Copulation behaviour of birds. Behaviour, 101, 101–138.

[ece39578-bib-0008] Bolnick, D. I. , & Fitzpatrick, B. M. (2007). Sympatric speciation: Models and empirical evidence. Annual Review of Ecology, Evolution, and Systematics, 38, 459–487.

[ece39578-bib-0009] Carpenter, B. , Gelman, A. , Hoffman, M. D. , Lee, D. , Goodrich, B. , Betancourt, M. , Brubaker, M. A. , Guo, J. , Li, P. , & Riddell, A. (2017). Stan: A probabilistic programming language. Journal of Statistical Software, 76. https://www.osti.gov/biblio/1430202 10.18637/jss.v076.i01 PMC9788645 36568334

[ece39578-bib-0010] Catry, P. , Phillips, R. A. , & Furness, R. W. (1999). Evolution of reversed sexual size dimorphism in skuas and jaegers. Auk, 116, 158–168.

[ece39578-bib-0011] Class, B. , & Brommer, J. E. (2018). Shared environmental effects bias phenotypic estimates of assortative mating in a wild bird. Biology Letters, 14, 20180106. 29997185 10.1098/rsbl.2018.0106 PMC6083224

[ece39578-bib-0012] Coyne, J. A. , & Orr, H. A. (2004). Speciation. Sinauer Associates, Inc.

[ece39578-bib-0013] Crespi, B. J. (1989). Causes of assortative mating in arthropods. Animal Behaviour, 38, 980–1000.

[ece39578-bib-0014] de Almeida Borghezan, E. , da Silva Pinto, K. , Zuanon, J. , & da Silva Pires, T. H. (2019). Someone like me: Size‐assortative pairing and mating in an Amazonian fish, Sailfin Tetra *Crenuchus spilurus* . PLoS One, 14, e0222880. 31560725 10.1371/journal.pone.0222880 PMC6764797

[ece39578-bib-0015] Elmer, K. R. , Lehtonen, T. K. , & Meyer, A. (2009). Color assortative mating contributes to sympatric divergence of neotropical cichlid fish. Evolution, 63, 2750–2757. 19490078 10.1111/j.1558-5646.2009.00736.x

[ece39578-bib-0016] Erlandsson, J. , & Rolán‐Alvarez, E. (1998). Sexual selection and assortative mating by size and their roles in the maintenance of a polymorphism in Swedish *Littorina saxatilis* populations. Hydrobiologia, 378, 59–69.

[ece39578-bib-0017] Fridolfsson, A. K. , & Ellegren, H. (1999). A simple and universal method for molecular sexing of non‐ratite birds. Journal of Avian Biology, 30, 116–121.

[ece39578-bib-0018] Galeotti, P. , & Rubolini, D. (2007). Head ornaments in owls: What are their functions? Journal of Avian Biology, 38, 731–736.

[ece39578-bib-0019] Galipaud, M. , Bollache, L. , & Dechaume‐Moncharmont, F. X. (2013). Assortative mating by size without a size‐based preference: The female‐sooner norm as a mate‐guarding criterion. Animal Behaviour, 85, 35–41.

[ece39578-bib-0020] Grant, P. R. , & Grant, B. R. (2008). Pedigrees, assortative mating and speciation in Darwin's finches. Proceedings of the Royal Society B: Biological Sciences, 275, 661–668. 10.1098/rspb.2007.0898 PMC2596835 18211884

[ece39578-bib-0021] Green, D. M. (2019). Rarity of size‐assortative mating in animals: Assessing the evidence with anuran amphibians. The American Naturalist, 193, 279–295. 10.1086/701124 30720359

[ece39578-bib-0023] Hakkarainen, H. , Lahti, K. , Huhta, E. , Lundvall, P. , Mappes, T. , Tolonen, P. , & Wiehn, J. (1996). A test of male mating and hunting success in the kestrel: The advantages of smallness? Behavioral Ecology and Sociobiology, 39, 375–380.

[ece39578-bib-0024] Han, C. S. , Jablonski, P. G. , Kim, B. , & Park, F. C. (2010). Size‐assortative mating and sexual size dimorphism are predictable from simple mechanics of mate‐grasping behavior. BMC Evolutionary Biology, 10, 359. 21092131 10.1186/1471-2148-10-359 PMC3003276

[ece39578-bib-0025] Hedenström, A. (1987). Assortative mating in the little ringed plover *Charadrius dubius* . Ornis Scandinavica, 18, 325–327.

[ece39578-bib-0026] Hendry, A. P. , & Day, T. (2005). Population structure attributable to reproductive time: Isolation by time and adaptation by time. Molecular Ecology, 14, 901–916. 15773924 10.1111/j.1365-294X.2005.02480.x

[ece39578-bib-0027] Indykiewicz, P. , Podlaszczuk, P. , Surmacki, A. , Kudelska, K. , Kosicki, J. , Kamiński, M. , & Minias, P. (2017). Scale‐of‐choice effect in the assortative mating by multiple ornamental and non‐ornamental characters in the Black‐headed Gull. Behavioral Ecology and Sociobiology, 71, 183.

[ece39578-bib-0028] Jiang, Y. , Bolnick, D. I. , & Kirkpatrick, M. (2013). Assortative mating in animals. The American Naturalist, 181, E125–E138. 10.1086/670160 23669548

[ece39578-bib-0029] König, C. , & Weick, F. (2008). Owls of the world. Yale University Press.

[ece39578-bib-0030] Krüger, O. (2005). The evolution of reversed sexual size dimorphism in hawks, falcons and owls: A comparative study. Evolutionary Ecology, 19, 467–486.

[ece39578-bib-0031] Lehmann, L. , Keller, L. F. , & Kokko, H. (2007). Mate choice evolution, dominance effects, and the maintenance of genetic variation. Journal of Theoretical Biology, 244, 282–295. 16979189 10.1016/j.jtbi.2006.07.033

[ece39578-bib-0032] Luo, S. (2017). Assortative mating and couple similarity: Patterns, mechanisms, and consequences. Social and Personality Psychology Compass, 11, e12337.

[ece39578-bib-0033] Lynch, M. , & Walsh, B. (1998). Genetics and analysis of quantitative traits. Sinauer.

[ece39578-bib-0034] Mantel, N. (1967). The detection of disease clustering and a generalized regression approach. Cancer Research, 27, 209–220. 6018555

[ece39578-bib-0035] Massemin, S. , Korpimäki, E. , & Wiehn, J. (2000). Reversed sexual size dimorphism in raptors: Evaluation of the hypotheses in kestrels breeding in a temporally changing environment. Oecologia, 124, 26–32. 28308409 10.1007/s004420050021

[ece39578-bib-0036] McDonald, P. G. , Olsen, P. D. , & Cockburn, A. (2005). Sex allocation and nestling survival in a dimorphic raptor: Does size matter? Behavioral Ecology, 16, 922–930.

[ece39578-bib-0037] Mueller, H. C. (1986). The evolution of reversed sexual dimorphism in owls: An empirical analysis of possible selective factors. Wilson Bull, 98, 387–406.

[ece39578-bib-0061] Murakami, R. , Sawada, A. , Ono, H. , & Takagi, M. (2022). The effect of experience on parental role Division in Ryukyu Scops Owl *Otus elegans* . Ornithological Science, 21(1). 10.2326/osj.21.35

[ece39578-bib-0039] Ornithological Society of Japan . (2012). Check‐list of Japanese birds, 7th revised edn. The Ornithological Society of Japan.

[ece39578-bib-0040] Owens, I. P. F. , & Hartley, I. R. (1998). Sexual dimorphism in birds: Why are there so many different forms of dimorphism? Proceedings of the Royal Society B: Biological Sciences, 265, 397–407.

[ece39578-bib-0041] Pande, S. , & Dahanukar, N. (2012). Reversed sexual dimorphism and differential prey delivery in Barn Owls (*Tyto alba*). Journal of Raptor Research, 46, 184–189.

[ece39578-bib-0042] Pincheira‐Donoso, D. , & Hunt, J. (2017). Fecundity selection theory: Concepts and evidence. Biological Reviews, 92, 341–356. 26526765 10.1111/brv.12232

[ece39578-bib-0043] Podos, J. (2010). Acoustic discrimination of sympatric morphs in Darwin's Finches: A behavioural mechanism for assortative mating? Philosophical Transactions of the Royal Society B, 365, 1031–1039. 10.1098/rstb.2009.0289 PMC2830235 20194166

[ece39578-bib-0044] R Core Team . (2021). R: A language and environment for statistical computing. R Foundation for Statistical Computing http://www.r‐project.org/

[ece39578-bib-0045] Sawada, A. , Ando, H. , & Takagi, M. (2020). Evaluating the existence and benefit of major histocompatibility complex‐based mate choice in an isolated owl population. Journal of Evolutionary Biology, 33, 762–772. 32281698 10.1111/jeb.13629

[ece39578-bib-0046] Sawada, A. , Iwasaki, T. , Inoue, C. , Nakaoka, K. , Nakanishi, T. , Sawada, J. , Aso, N. , Nagai, S. , Ono, H. , & Takagi, M. (2021). Missing piece of top predator‐based conservation: Demographic analysis of an owl population on a remote subtropical Island. Population Ecology, 63, 204–218.

[ece39578-bib-0047] Sawada, A. , Iwasaki, T. , Matsuo, T. , Akatani, K. , & Takagi, M. (2021). Reversed sexual size dimorphism in the Ryukyu Scops Owl *Otus elegans* on Minami‐Daito Island. Ornithological Science, 20, 15–26.

[ece39578-bib-0048] Sawada, A. , Iwasaki, T. , & Takagi, M. (2019). Fine‐scale spatial genetic structure in the Minami‐Daito Island population of the Ryukyu Scops Owl *Otus elegans* . Journal of Zoology, 307, 159–166.

[ece39578-bib-0049] Schoenjahn, J. , Pavey, C. R. , & Walter, G. H. (2020). Why female birds of prey are larger than males. Biological Journal of the Linnean Society, 129, 532–542.

[ece39578-bib-0050] Stan Development Team . (2018). Stan modeling language users guide and reference manual, version 2.18.0. http://mc‐stan.org

[ece39578-bib-0051] Stan Development Team . (2019). RStan: the R interface to Stan. R package version 2.19.2. 016. http://mc‐stan.org

[ece39578-bib-0052] Taborsky, B. , Guyer, L. , & Taborsky, M. (2009). Size‐assortative mating in the absence of mate choice. Animal Behaviour, 77, 439–448.

[ece39578-bib-0053] Takagi, M. (2020). Vocalizations of the Ryukyu Scops Owl *Otus elegans*: Individually recognizable and stable. Bioacoustics, 29, 28–44.

[ece39578-bib-0054] Takagi, M. , & Akatani, K. (2011). The diet of Ryukyu Scops Owl *Otus elegans* interpositus owlets on Minami‐Daito Island. Ornithological Science, 10, 151–156.

[ece39578-bib-0055] Takagi, M. , Akatani, K. , Matsui, S. , & Saito, A. (2007). Status of the Daito Scops owl on Minami‐Daito Island, Japan. Journal of Raptor Research, 41, 52–56.

[ece39578-bib-0056] Wagner, R. H. (1999). Sexual size dimorphism and assortative mating in Razorbills (*Alca torda*). Auk, 116, 542–544.

[ece39578-bib-0057] Wang, D. , Forstmeier, W. , Valcu, M. , Dingemanse, N. J. , Bulla, M. , Both, C. , Duckworth, R. A. , Kiere, L. M. , Karell, P. , Albrecht, T. , & Kempenaers, B. (2019). Scrutinizing assortative mating in birds. PLoS Biology, 17, e3000156. 30789896 10.1371/journal.pbio.3000156 PMC6400405

[ece39578-bib-0058] Warkentin, I. G. , James, P. C. , & Oliphant, L. W. (1992). Assortative mating in urban‐breeding Merlins. Condor, 94, 418–426.

[ece39578-bib-0059] Wojczulanis‐Jakubas, K. , Drobniak, S. M. , Jakubas, D. , Kulpińska‐Chamera, M. , & Chastel, O. (2018). Assortative mating patterns of multiple phenotypic traits in a long‐lived seabird. IBIS, 160, 464–469.

[ece39578-bib-0060] Zou, G. Y. (2007). Toward using confidence intervals to compare correlations. Psychological Methods, 12, 399–413. 18179351 10.1037/1082-989X.12.4.399

