# Supplementary material for: Mate choice for body size leads to size assortative mating in the Ryukyu Scops Owl Otus elegans
Source: Ecol Evol. 2022 Dec 12;12(12):e9578. doi: 10.1002/ece3.9578 (PMC9745103; doi:10.1002/ece3.9578)
Supplement: Supplementary file 9 — Appendix S1 [file ECE3-12-e9578-s009.docx]

**Figure Legends**

Figure S1 Correlation between traits in males. Correlation coefficients ranged from −0.466 to 0.453. Calculation was based on data of 331 individuals used for analysis of fitness components.

Figure S2 Scatter plot of body measurements of males and females in mated pairs based on first measurements. For the traits which have p-values below 0.05 in a parametric test for Pearson's correlation, estimated regression lines are depicted.

Figure S3 Parent–offspring regressions of (a) culmen length and (b) wing length.

Figure S4 Scatter plot of body measurements of males and females in mated pairs based on last measurements. For traits with p-values below 0.05 in parametric tests for Pearson's correlation, estimated regression lines are depicted.

Figure S5 Conceptual diagram of data standardization for effect of measurers. This diagram shows difference between measurement values of culmen length by measure KA, TI, and AS. By fitting measurement data to regression model like linear model, systematic differences between measures are estimated as this. Therefore, raw data can be controlled for the effects of measure by properly adding or subtracting these difference estimates. In this study we refer to Sawada et al. (2021b) for these difference estimates.

Figure S6 The reason why raw data should be standardized before calculation of correlation coefficient for assessment of assortative mating. These graphs are hypothetical scatterplots of culmen length (correspond to Figure S5). Since there are systematic differences in measurement values between measurers (KA, TI, and AS), the distribution of raw data become long-stretched (a), compared with the distribution of standardized data (b). If we ignore it, strong correlation relationship is estimated just due to effect of measurer.

Figure S7 Data matrix and on which analyses were based.
